# Aberrant connectivity in the hippocampus, bilateral insula and temporal poles precedes treatment resistance in first-episode psychosis: a prospective resting-state functional magnetic resonance imaging study with connectivity concordance mapping

**DOI:** 10.1093/braincomms/fcae094

**Published:** 2024-05-04

**Authors:** Stavros Skouras, Maria-Lisa Kleinert, Edwin H M Lee, Christy L M Hui, Yi Nam Suen, Jazmin Camchong, Catherine S Y Chong, Wing Chung Chang, Sherry K W Chan, William T L Lo, Kelvin O Lim, Eric Y H Chen

**Affiliations:** Department of Fundamental Neurosciences, Faculty of Medicine, University of Geneva, CH-1211 Geneva, Switzerland; Department of Neurology, Inselspital University Hospital Bern, CH3010 Bern, Switzerland; Faculty of Medicine, University of Barcelona, 08036 Barcelona, Spain; Department of Psychiatry, University of Hong Kong, Hong Kong, China; Department of Psychiatry, University of Hong Kong, Hong Kong, China; Department of Psychiatry, University of Hong Kong, Hong Kong, China; Department of Psychiatry, University of Minnesota, Minneapolis, MN 55454, USA; Department of Psychiatry, Kwai Chung Hospital, Hong Kong, China; Department of Psychiatry, University of Hong Kong, Hong Kong, China; Department of Psychiatry, University of Hong Kong, Hong Kong, China; Department of Psychiatry, Kwai Chung Hospital, Hong Kong, China; Department of Psychiatry, University of Minnesota, Minneapolis, MN 55454, USA; Department of Psychiatry, University of Hong Kong, Hong Kong, China

**Keywords:** treatment resistant, schizophrenia, resting-state, fMRI, connectivity concordance mapping

## Abstract

Functional connectivity resting-state functional magnetic resonance imaging has been proposed to predict antipsychotic treatment response in schizophrenia. However, only a few prospective studies have examined baseline resting-state functional magnetic resonance imaging data in drug-naïve first-episode schizophrenia patients with regard to subsequent treatment response. Data-driven approaches to conceptualize and measure functional connectivity patterns vary broadly, and model-free, voxel-wise, whole-brain analysis techniques are scarce. Here, we apply such a method, called connectivity concordance mapping to resting-state functional magnetic resonance imaging data acquired from an Asian sample (*n* = 60) with first-episode psychosis, prior to pharmaceutical treatment. Using a longitudinal design, 12 months after the resting-state functional magnetic resonance imaging, we measured and classified patients into two groups based on psychometric testing: treatment responsive and treatment resistant. Next, we compared the two groups’ connectivity concordance maps that were derived from the resting-state functional magnetic resonance imaging data at baseline. We have identified consistently higher functional connectivity in the treatment-resistant group in a network including the left hippocampus, bilateral insula and temporal poles. These data-driven novel findings can help researchers to consider new regions of interest and facilitate biomarker development in order to identify treatment-resistant schizophrenia patients early, in advance of treatment and at the time of their first psychotic episode.

## Teaser

Skouras *et al*. used concordance connectivity mapping, an advanced method of functional connectomics, that highlights differences in the consistency of functional connectivity between two groups. Results show that prior to medication, treatment-resistant patients with first-episode psychosis show more consistent functional connectivity patterns in the hippocampus, insula and temporal poles.

## Graphical Abstract

**Graphical Abstract:**
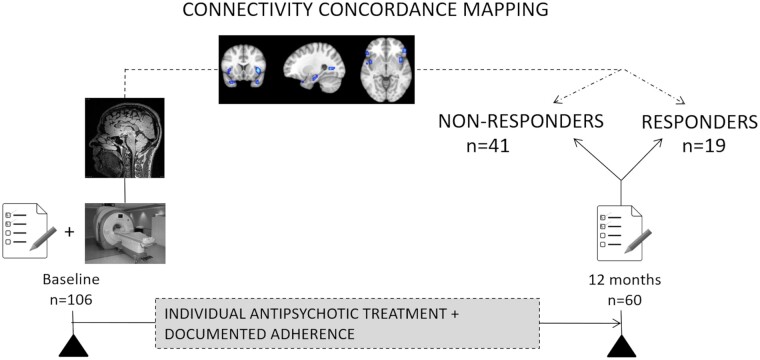

## Introduction

Schizophrenia is a lifelong and severely disabling neurodevelopmental disorder,^1^ with a lifetime prevalence of ∼0.7% worldwide.^2^ The course of schizophrenia can follow a heterogeneous range of trajectories, from severe cases of repeated relapse and deterioration, to cases in which a single psychotic episode is followed by complete recovery.^3^ Antipsychotic pharmacotherapy continues to be the frontline treatment for schizophrenia^4,5^; however, an integrated treatment approach should always consider psychosocial interventions and attention to environmental circumstances.^3^ A promising window for course-altering interventions is the time of initial diagnosis of the first-episode psychosis (FEP).^6^ Rapid intervention with antipsychotics decreases the duration of untreated psychosis and, therefore, enhances patients’ quality of life, social functioning and long-term outcomes.^7^

Nevertheless, about 30–35% of patients with schizophrenia do not respond to first-line antipsychotics and therefore fall into the category of treatment-resistant schizophrenia (TRS).^8^ Other studies reported this proportion of patients to be even higher, in the range of 40–60%.^9,10^ Treatment-resistant patients tend to experience more persistent positive, negative and cognitive symptoms, such as wide-ranging deficits in verbal memory and learning and in language functions,^11^ leading to worsened social functioning, including lower marriage rates and increased likelihood of residence in facilities.^12^ Furthermore, treatment resistance is associated with a higher socio-economic burden,^13^ long-term disability^12,13^ and higher family/caregiver burden.^14^

TRS can be defined in many ways and comparing studies of TRS can be ‘akin to comparing apples to oranges’.^15^ This leads to inconsistent and not evidence-based identification and management of TRS in clinical practice.^16^ As a result, consensus criteria have been proposed to define TRS as characterized by a limited symptom reduction [as assessed by e.g. the Positive and Negative Syndrome Scale (PANSS) or the Brief Psychiatric Rating Scale (BPRS)] with at least moderate functional impairment during a prospective trial or observation of ≥6 weeks and ≥2 past adequate treatment episodes with different non-clozapine antipsychotic drugs.^15,16^

Therapeutic nihilism can result in sustained ineffective treatment, the delay of appropriate treatment or not offering it at all.^17^ Having prior knowledge of patients’ antipsychotic treatment response opens up several avenues for improving clinical decision-making and, consequently, patient outcomes.^18^ Clozapine is considered the first-line pharmacological agent for patients with TRS. Second-line treatment options include pharmacological and non-pharmacological augmentation of clozapine, such as medication combinations, electroconvulsive therapy (ECT), repetitive transcranial magnetic stimulation, deep brain stimulation and cognitive behavioral therapy.^19^ Early identification of TRS is key because 40–50% of these patients respond to clozapine.^20,21^ Furthermore, the shorter the delay of clozapine initiation, the better the response to it.^22,23^

Since clozapine exhibits low dopamine receptor D2 occupancy compared with other antipsychotics, clozapine’s success in treating TRS patients suggests that its underlying mechanism of action may extend beyond dopamine receptors.^24^ Aberrant interactions between dopaminergic and glutamatergic signalling pathways in individuals with TRS have been proposed.^25-27^ In this scenario, an *N*-methyl-D-aspartate receptor hypofunction leads to decreased activity of cortical GABA-ergic interneurons and a diminished inhibitory control of pyramidal glutamate neurons, which accounts for increased activity of dopaminergic projections from the midbrain to the striatum.^28^ The pharmacodynamics of clozapine include its ability to modulate glutamate activity. Clozapine’s hypothesized effects involve partial agonist activity of *N*-methyl-D-aspartate receptor, along with its ability to regulate glutamate transport, leading to a potential enhancement of glutamate transmission.^29-31^ However, it is probably the collective impact of clozapine’s affinity to a diverse range of neuroreceptors, e.g. D_4_, 5-HT_2A_, α_1_ and m_1_, that explains its superior effectiveness compared with classical antipsychotics in the setting of TRS. This aberrant synaptic connectivity, leading to a disruption of communication between brain networks, embodies the cornerstone within the disconnection hypothesis of the psychopathology of schizophrenia,^32^ although it is worth noting that aberrant glutamatergic signalling pathways linked to hyperdopaminergic states are not specific to TRS and are possible in all disorders within the psychosis spectrum. Moreover, there is evidence suggesting that TRS in FEP might be neurobiologically different from TRS developing over time.^33,34^ Whereas the former features normal dopamine function, while glutamate or other pathways contribute to the distinct neurobiology of treatment resistance,^34^ the latter can be explained by progressive dopamine hypersensitivity in the striatum induced by continuous antipsychotic dopamine receptor D2 blockage.^35^ Both models are not mutually exclusive but could explain the neurobiological differences between two clinically different presentations of TRS, i.e. those who do not respond from the beginning and those who respond initially but their response wears off after some time.^34^

Functional connectivity (FC) analysis of resting-state functional MRI (rs-fMRI) measures temporal correlations of spontaneous blood oxygen levels (BOLD) amongst spatially distributed brain regions, with the assumption that areas with correlated activity form functional networks.^36^ Recent evidence suggests that FC during rs-fMRI holds promise as a predictive biomarker for TRS.^18,23,37^ Disrupted FC between different brain regions has been associated with TRS, specifically involving the prefrontal cortex, striatum and other subcortical structures,^38^ the insula and the anterior cingulate cortex^39^ and the central opercular cortex and the sensorimotor cortex.^40^ In a systematic review of rs-fMRI investigations for predicting antipsychotic response in schizophrenia, Mehta *et al*.^18^ reported striatal and default mode network functional segregation and integration metrics to be consistent determinants of treatment response (with a pooled odds ratio of 12.66). Nevertheless, research has not yet converged on any specific hypothesis of aberrant FC that distinguishes treatment responders from non-responders.^40^

The lack of consensus is favoured by several issues. On the one hand, to date, most studies that examine FC rs-fMRI compare responders and non-responders to antipsychotic treatment on a cross-sectional level (see recent review on such cross-sectional studies^41^). Not only can the results of such studies be considered biased by the type of antipsychotic treatment itself but also comparisons are often made between non-responders and healthy controls, instead of non-responders versus responders. Only few prospective studies exist that examine baseline rs-fMRI data in FEP patients with regard to subsequent treatment response over a time period of weeks/months. On the other hand, the lack of consensus might be a consequence of the broad variety of data analytical approaches that have been used to conceptualize and measure FC. For instance, some researchers used the specific FC of two *a priori* regions of interest (ROIs) with eight other *a priori* ROIs.^42^ Others measured FC as the co-activation of *a priori* seed ROIs with the rest of the brain.^43^ Independent component analysis relies on *post hoc* decisions in order to determine which components are functionally relevant, by measuring the co-activation between brain networks with the rest of the brain.^44,45^ With few exceptions, graph theory approaches typically examine pairwise co-activations between all ROIs defined by anatomical and functional parcellation units from a brain atlas.^46^ What most approaches have in common is that they are driven by specific hypotheses about regions or networks of interest, which could bias results and conclusions. Furthermore, the variety of analytical algorithms may very well measure distinct underlying phenomena^8^ and does not facilitate the convergence of findings across studies.^38^ Thereby, it is crucial to use voxel-wise, whole-brain algorithms to allow bias-free neuromarker discovery for TRS in FEP patients. To our knowledge, the present study is the first to use connectivity concordance mapping (CCM) in this context. CCM is a computationally intensive method that identifies the brain areas that exhibit the highest consistency in patterns of FC, across subjects (see ‘Materials and methods’). The result of a CCM analysis is a voxel-wise map of concordance values. Regions of high inter-subject concordance can be assumed to be functionally consistent and may thus be used as ROIs for further investigations.^47^

## Materials and methods

### Participants

This study was approved by the Ethics Committee of the Institutional Review Board of the University of Hong Kong/Hospital Authority Hong Kong West Cluster. Written informed consent was obtained from all the participants (or their parents for those under the age of 18 years) after complete description of the study. The study was performed in accordance with the Declaration of Helsinki. Between July 2014 and July 2017, a total of 106 patients aged between 15 and 25 years with a FEP were recruited from the Early Assessment Service for Young people with psychosis in Hong Kong, which provides a specialized intervention service to FEP. Patients were selected consecutively from all in- and out-patient psychiatric departments at Queen Mary Hospital and Kwai Chung Hospital.

Patients with a DSM-IV diagnosis of schizophrenia, schizophreniform disorder, schizoaffective disorder, brief psychotic disorder, delusional disorder or psychosis not otherwise specified were included in the study. Participants had to be first-episode cases with no antipsychotic treatment history. Exclusion criteria were intellectual disability, substance-induced psychosis, psychotic disorder because of a general medical condition or an inability to speak Cantonese Chinese for the research interview.

All patients underwent MRI scanning at baseline. The study followed a naturalistic observational study design with no influence on the following therapy and clinical assessments determined by the patients’ clinicians that were performed according to standard clinical practice. The majority of patients received second-generation antipsychotics in doses recommended by APA Practice Guidelines. Chlorpromazine equivalent doses were computed for analysis (50–1000 mg/day).^48^ Out of the 106 participants who entered the study and received the baseline assessment including rs-fMRI scans, only 60 participants could be followed up at 12 months due to a high drop-out rate of 43%.

### Study design

**Figure 1:**
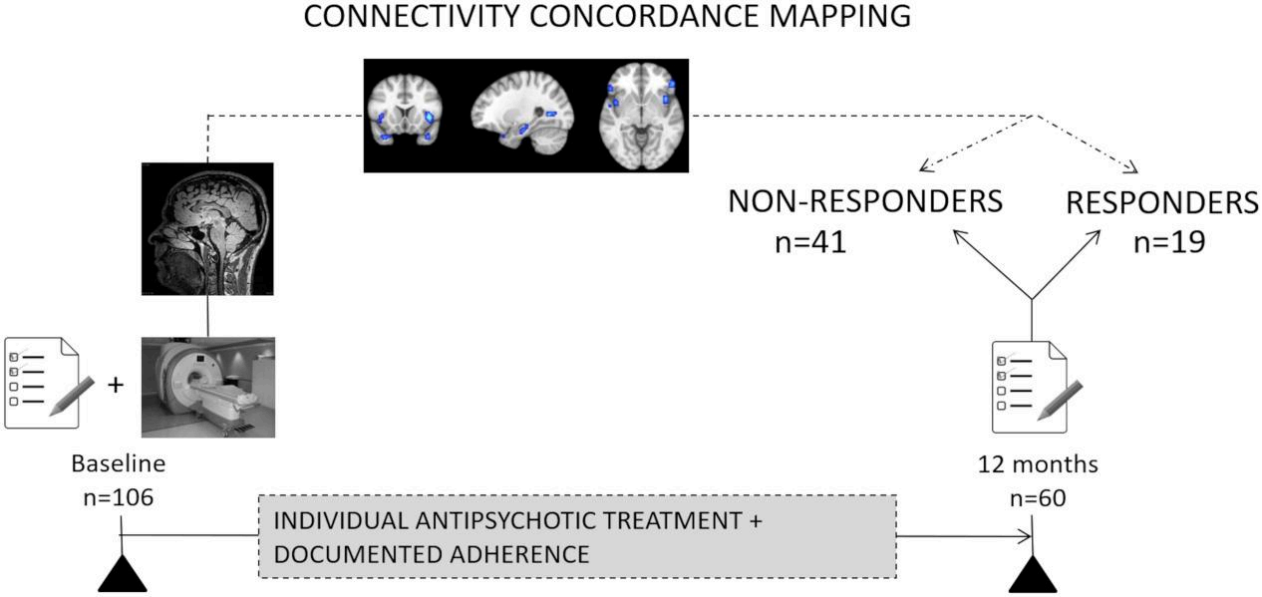
**Study design.** A total of 106 patients with a FEP were recruited and underwent clinical, functional and neurocognitive assessments at baseline, including anatomical and functional MRI. Over the course of the following 12 months, participants received their individual antipsychotic treatment. Sixty participants participated in follow-up visits after 12 months due to a high drop-out rate. At that time, we classified the remaining participants into responders (*n* = 19) and non-responders (*n* = 41), based on their longitudinal neurocognitive assessments. In order to qualify as a non-responder, the following criteria had to be met, in relation to the baseline assessments: <50% symptom reduction in total PANSS score, at least moderate functional impairment assessed with Social and Occupational Functioning Assessment Scale score and ≥2 past adequate treatment episodes with different non-clozapine antipsychotic drugs. To investigate patterns of brain function that relate to responsiveness to antipsychotic medication, we applied CCM to the rs-fMRI data acquired at baseline and compared the connectivity concordance maps of the two groups. See also Supplementary Table 1.

The study was performed as a prospective, 1-year follow-up study of treatment response in FEP patients. As shown in Fig. 1, assessments took place at the first intake of antipsychotic treatment and 12 months later. Basic demographics, premorbid functioning and course-related variables were assessed at study entry. Other clinical, functional and neurocognitive predictors were assessed at baseline and at 12 months. See Supplementary Table 1 for a complete list of measurements and questionnaires used. For the purposes of this study, anatomical and functional MRI data were collected in all patients at baseline.

### Responders versus non-responders classification criteria and group characteristics

Treatment responsiveness was assessed with the PANSS^49^ by experienced psychiatrists. The following criteria were adopted to define TRS: <50% symptom reduction in total PANSS score and at least moderate functional impairment assessed with Social and Occupational Functioning Assessment Scale score, 12 months after FEP and ≥2 past adequate treatment episodes with different non-clozapine antipsychotic drugs and documented adherence. According to these criteria, participants were divided into two groups: treatment responsive (*n* = 19) and treatment resistant (*n* = 41). Table 1 shows the demographic and clinical data of both groups.

**Table 1:** Demographical and clinical data of participants

	Responders (*n* = 19)	Non-responders (*n* = 41)	Statistical testing
Age (y)	26.3 ± 13.2	28.3 ± 12.0	*t*(58) = 0.804
			*P* = 0.432
Gender (M/F)	7/12	13/28	*χ* ^2^(1, 60) = 0.154
			*P* = 0.695
Education level (y)	12.1 ± 3.2	11.8 ± 3.7	*t*(58) = 0.794
			*P* = 0.435
DUP (d)	220 ± 314	285 ± 458	*t*(58) = 0.241
			*P* = 0.819
PANSS score at baseline	53.68 ± 13.85	39.12 ± 6.66	*t*(58) = 5.2
			*P* < 0.001
Antipsychotic dose (mg/d)^a^	403.6 ± 267.1	367.4 ± 176.0	*t*(58) = −0.041
			*P* = 0.968

DUP, duration of untreated psychosis; PANSS, Positive and Negative Syndrome Scale.

^a^Dose of antipsychotic medication at 12 m was converted to chlorpromazine equivalent dosages as proposed by Woods.^48^

### MRI data acquisition

MRI data were acquired on a 3.0-T Philips Achieva whole-body MRI scanner (Philips, The Netherlands), at the University of Hong Kong. A high-resolution (1 × 1 × 1 mm) T_1_-weighted anatomical reference image was acquired from each participant using a rapid acquisition gradient echo sequence featuring 160 slices per volume. During rs-fMRI, continuous echo planar imaging was used with an echo time of 30 ms and a repetition time of 2 s. Slice acquisition was interleaved within the repetition time interval. Two hundred fifty whole-brain volumes were acquired for each participant. The matrix acquired was 80 × 80 voxels with a field of view of 240 mm, resulting in an in-plane resolution of 3 mm. Slice thickness was 3.5 mm (38 slices, whole-brain coverage).

### Image processing

#### Anatomical images

T_1_ images were processed with the N4 nonparametric non-uniform intensity normalization bias correction function^50^ of the Advanced Normalization Tools^51^ and an optimized blockwise non-local means denoising filter.^52^ To segment anatomical images into grey matter, white matter and CSF, the VBM8 (Structural Brain Mapping Group, University of Jena, Jena, Germany; http://www.neuro.uni-jena.de/vbm/) and SPM12 (Wellcome Department of Imaging Neuroscience Group, London, UK; http://www.fil.ion.ucl.ac.uk/spm) toolboxes were used. The cranium was accurately removed from anatomical brain images using graph-cut^53^ and FSL (FMRIB, Oxford, UK; https://fsl.fmrib.ox.ac.uk/fsl/). A custom anatomical template was computed for the entire sample using the Advanced Normalization Tools multivariate template construction process.^54,55^ We computed neuroanatomically plausible symmetric diffeomorphic matrices in order to transform each subject’s anatomical data to the optimal template and afterwards to the Montreal Neurological Institute (MNI) space^54,56^ as defined by the Chinese brain atlas ‘Chinese_56’.^57^ Prior to the normalization of data sets and similarly as for the anatomical data, the cranium of the Chinese_56 brain atlas had also been removed. In accordance with best practices as well as to ensure optimal normalization while avoiding multiple interpolations, all transformation matrices were concatenated and applied to the data sets in a single step.

#### Functional images

Using Matlab 2014b (MathWorks Inc., Natick, MA, USA), SPM12 and the CONN functional connectivity toolbox (v17; www.conn-toolbox.org), functional data were processed according to the following steps: slice time correction, estimation of movement parameters, co-registration, band-pass filtering between 0.1 and 0.01 Hz, detrending, denoising and repairing of artefacts using the 95th percentile settings of the ART artefact detection tools. Only subjects with at least 90% valid volumes were considered further in the analysis and ‘scan nulling’ regressors were applied on any affected volumes.^58^ Average CSF signal, average white matter signal and 24 Volterra expansion movement parameters were regressed out of each participant's time series. Using FSL, each subject’s functional data was masked by their equivalent grey matter masks. Using Advanced Normalization Tools, the functional data were normalized to MNI space based on their respective diffeomorphic matrices. Functional data sets were smoothed by a 6 mm FWHM kernel, using the Leipzig Image Processing and Statistical Inference Algorithms (version 2.2.7).

### Statistical analysis and CCM algorithm

Chi-square goodness-of-fit testing was used to assess the normality of the distribution of each potential confounding variable for our sample. For the variables where the assumption of normality was violated, we utilized non-parametric independent samples *t*-tests, with 100 000 permutations per test (that do not require the parametric assumptions to be met), to compare between the two groups in our sample (see results in Table 1).

Lohmann *et al*.^47^ proposed CCM as a tool for model-free analysis of rs-fMRI data of the human brain. The aim of CCM is to visualize the reproducibility or inter-subject consistency of FC patterns across the entire brain. For this purpose, we computed the correlations of time courses of each voxel with every other voxel in each data set. The result is a correlation pattern of each patient’s brain. In a second step, we looked at how consistent these correlation patterns are across patients within each group. Inter-subject consistency or concordance was measured using Kendall’s *W*.^59^ Voxels whose correlation pattern was consistent across all data sets within a patient group (e.g. non-responders) received high values, i.e. high concordance values. The result of a CCM analysis is a voxel-wise map of concordance values ranging from 0 to 1 (0 = no consistency across data sets; 1 = perfect consistency across data sets). For the purpose of this study, patients were divided into two groups according to the TRS criteria outlined above: treatment responsive (*n_a_* = 19) and treatment resistant (*n_b_* = 41). A connectivity concordance map was computed for each of the two groups separately, based on ∼50 000 grey matter voxels, by utilizing the CCM algorithm described by Lohmann *et al*.^47^

**Figure 2:**
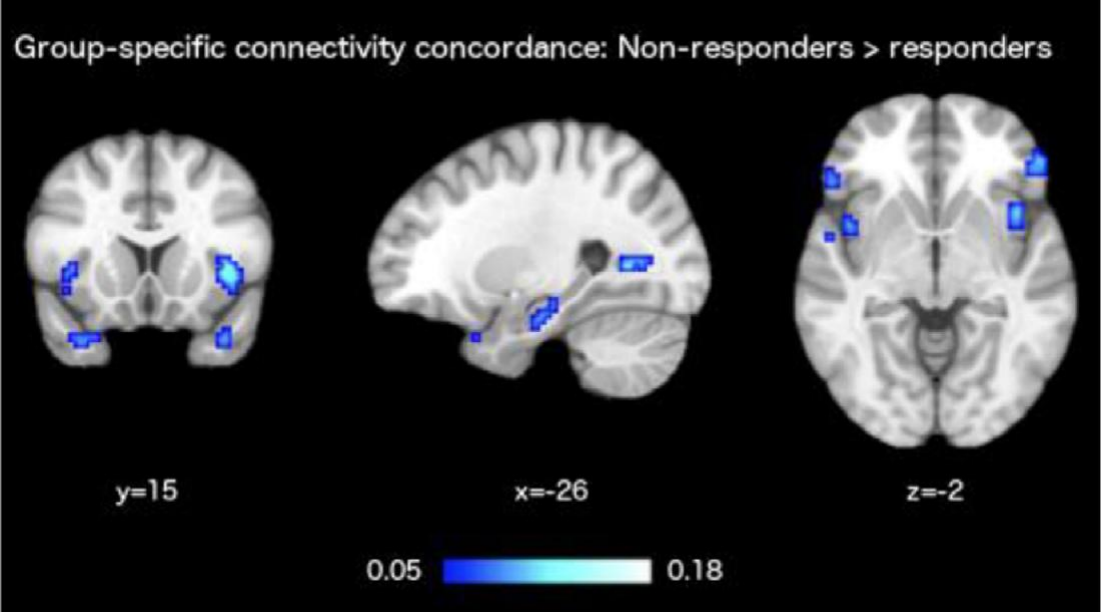
**fMRI results.** To investigate patterns of brain function that relate to responsiveness to antipsychotic medication, CCM was applied to rs-fMRI data acquired data from schizophrenia patients soon after the time of FEP. Two CCM images were computed: one for the group of responders and one for the group of non-responders. Their subtraction resulted in the presented second-level contrast image that depicts areas with higher connectivity concordance in patients who were classified as non-responders (i.e. treatment-resistant patients). The colour map shows the magnitude of differences between the two groups, in CCM values based on Kendall’s *W*, particularly in the hippocampus, bilateral insula and temporal poles. Kendall’s *W* is a statistical coefficient of concordance that assumes voxel values in the range between 0 (indicating no consistency of FC across data sets in the group) and 1 (indicating perfect consistency of FC across data sets in the group).

For each patient data set *k* and each voxel address *i*, a connectivity vector was obtained, whose $s_{i}^{k}=(s_{i,1}^{k},\;\ldots ,\;s_{i,n}^{k})$ entries $s_{i,j}^{k}$ contain a pairwise similarity measure between the time courses of voxels *i* and *j*. In this study, the pairwise similarity measure used was Pearson’s linear correlation coefficient, defined as follows:


$$r_{xy}=\;\frac{\sum_{t}\;(x_{t}-\;\bar{x})(y_{t}-\;\bar{y})}{\sqrt{\sum_{t}\;{\left(x_{t}-\;\bar{x}\right)}^{2}{\left(y_{t}-\;\bar{y}\right)}^{2}}},$$

with *x_t_* and *y_t_* being the time series in a pair of two voxels *x* and *y*, and $\bar{x},\;\bar{y}$ being their temporal means. The point of interest in CCM is the concordance of the connectivity vectors $s_{i}^{k}$ across the multiple patient data sets of each group. Kendall’s *W*, which is a non-parametric statistic for rank correlation, was used as a measure of concordance. It yields values between 0 (total disagreement) and 1 (total agreement) across data sets, without a requirement for any parametric assumptions to be met. Via a resource-intensive computational process, one such concordance value is computed across all the data sets of each group while considering the pairwise similarity of each voxel with all other voxels, for each of ∼50 000 grey matter voxels. This concordance value becomes the CCM in voxel *I*, resulting in two images: one CCM image for the treatment-responsive group and one CCM image for the treatment-resistant group. Each of these images quantified how consistent the connectivity pattern of each voxel was, across the participants belonging to the respective group. Lastly, the CCM image of the treatment-resistant group was subtracted from the CCM image of the treatment-responsive group, to obtain the difference in CCM values between the two groups (Δ_CCM_). Thresholding of Δ_CCM_ > 0.05 combined with a thresholding of cluster size *k* > 10 voxels was applied to produce the final results displayed in Fig. 2.

## Results

### Basic demographic and clinical characteristics

Table 1 shows the demographic information and clinical characteristics of our final sample (*N* = 60). Thirty-two per cent of the participants responded to antipsychotic treatment, whereas the remaining 68% were classified as treatment resistant. The two groups did not significantly differ in terms of age, gender, education, duration of untreated psychosis and antipsychotic medication dosages. However, there was a significant difference with regard to the PANSS score at baseline [*t*(58) = 5.2, *P* < 0.001, (confidence interval 8.7–19.5)]. The treatment-responsive group had a higher PANSS score at baseline (53.68 ± 13.85) compared to non-responders (39.12 ± 6.66).

### FC and connectivity concordance maps

Our analyses yielded the clusters depicted in Fig. 2 that show areas of more consistent FC for the treatment-resistant group, in a network including the left hippocampus, the bilateral insula and the temporal poles. Complete details are presented in Table 2.

**Table 2:** Areas with higher connectivity concordance in non-responders

AAL label	Hemisphere	MNI coordinates			Volume (mm^3^)	mean Δ_CCM_	max Δ_CCM_	SD Δ_CCM_
		*X*	*Y*	*Z*				
Supramarginal gyrus	Left	−51	−39	28	4212	0.07	0.12	0.01
BA13, pars opercularis	Right	39	15	4	1539	0.07	0.12	0.02
BA47	Left	−54	27	−5	1296	0.06	0.09	0.01
BA20, inferior temporal lobule	Left	−57	−9	−35	1080	0.08	0.19	0.03
Insula	Left	−33	24	7	891	0.06	0.08	0.01
Insula	Left	−45	12	−5	783	0.06	0.09	0.01
No label available (nearest to lingual gyrus)	Left	−27	−63	7	621	0.07	0.1	0.01
BA38, temporal pole	Right	39	18	−29	459	0.06	0.09	0.01
IFG, pars triangularis	Right	54	39	−2	405	0.06	0.08	0.01
Hippocampus	Left	−27	−21	−17	378	0.06	0.07	0.01
IFG, pars triangularis	Right	51	27	22	324	0.06	0.07	0.01

MNI coordinates specify the peak coordinate of each cluster. Labels correspond to the peak coordinates, according to the Automated Anatomical Labeling atlas and the xjview version 10.0 visualization tool (www.alivelearn.net/xjview/). The mean, max and standard deviation (SD) of each cluster are displayed.

AAL, Automated Anatomical Labelling; BA, Brodmann area; CCM, concordance connectivity mapping; IFG, inferior frontal gyrus; MNI, Montreal Neurological Institute.

## Discussion

The purpose of this study was to utilize an advanced data-driven, voxel-wise, whole-brain analysis approach to investigate rs-fMRI data from drug-naïve FEP patients, in order to discover potential ROIs to guide future research of TRS biomarkers. We identified a network of areas showing consistently increased FC, including the left hippocampus, bilateral insula and temporal poles in the treatment-resistant group. These findings are supported by previous studies that have linked TRS to increased FC in cortical and subcortical networks including the insula^60,61^ and the temporal poles.^60^ However, other studies have associated treatment resistance in schizophrenia patients with decreased FC of those same regions^62,63^ as well as the hippocampus.^64^ This highlights the relevance of our findings in relation to an ongoing debate. Our results suggest that these areas differ most between treatment-resistant and treatment-responsive patients, in terms of their connectivity with the rest of the brain, thereby explaining why previous studies have generated conflicting results.

In schizophrenia patients, the insula has been proposed as a potential brain area that may shed light on the neurobiological underpinnings of treatment resistance.^39^ Some of the various functions the insula is involved in include homeostasis,^65-67^ taste perception,^68,69^ auditory perception,^70,71^ multimodal sensory processing,^72^ pain,^73^ interoception,^74-76^ self-consciousness^77-79^ and social emotions^80,81^ This broad range of functions ensues from (i) extensive viscerosensory inputs into the region and (ii) the insula’s anatomical location that allows for strong reciprocal connections with the prefrontal, somatosensory and temporal areas, as well as with the limbic system.^82^ The anterior insula and the anterior cingulate cortex form the salience network. The salience network modulates both bottom-up and top-down processing of stimuli and, moreover, controls the switch from a resting-state and the default mode network to an attentive state and the central executive network.^83^ Altered auditory evoked potentials and abnormal sensory gating are key findings that help to explain auditory hallucinations, one of the most common positive symptoms found in schizophrenia patients.^84,85^ Strikingly, Alonso-Solís *et al*.^60^ found treatment-resistant auditory verbal hallucinations linked to higher FC in a network including the insular cortex. Likewise, alterations across various interoceptive systems have been identified in schizophrenia.^86^ Furthermore, aberrant activation and FC of the salience network^39,87^ and the default mode network^16,84^ have been associated with TRS. Interestingly, inter-individual diversity in the insula’s FC explains variability in some of the clinical symptoms of schizophrenia.^88^

Structural and functional abnormalities of the hippocampus are amongst the most consistent findings in schizophrenia neuroscience research.^89^ Hippocampal dysfunction may therefore explain two of the most prominent features of the schizophrenia clinical spectrum, i.e. cognitive deficits (including memory function) and reality distortion.^90,91^ A systematic review suggested that hippocampal hyperactivity (measured by BOLD, cerebral blood volume and cerebral blood flow) is caused by a decrease in hippocampal inhibitory interneurons in schizophrenia patients.^92^ In fact, hippocampal hyperactivity is one the most consistent functional aberrations in schizophrenia and it predates the onset of psychosis and hippocampal volume reduction.^92,93^ Interestingly, post-mortem studies heavily support a reduction in hippocampal subfield volumes, total neuron counts and neuron size but preserved neuron density, predominantly lateralized to the left hemisphere of schizophrenia patients.^94^ Moreover, these changes seem to be accentuated in TRS. When compared with matched treatment-responsive patients, TRS patients showed poorer performance in working memory and smaller hippocampal volume.^95^ The hippocampus can be considered as a cornerstone of the glutamate hypothesis of schizophrenia; hence, *N*-methyl-D-aspartate receptor hypofunction and decreased inhibitory interneuron activity support the idea of hippocampal hyperactivity. Dysfunctional connectivity, including connections to the striatum and the frontal lobes, could result from such interneuron abnormalities.^96^

Additional evidence for the hippocampus’ and the insula’s involvement in TRS comes from research in the field of ECT. There is growing support for the addition of ECT to antipsychotic treatment regimes in TRS.^97-99^ A recent systematic review of the neural effects of ECT in schizophrenia patients found the hippocampus and insula to be key regions of modulation after ECT.^100^ This applied to morphometry, FC and symptom association measures. The temporal pole is an integral component of the paralimbic circuit, together with the insula and the orbitofrontal cortex.^101^ The temporal pole plays an important role in various cognitive functions, olfaction and affectional–sensory integration.^102^ Abnormal connectivity patterns have been reported for the temporal pole as well as other limbic and paralimbic regions in schizophrenia.^103^ Goswami *et al.*^104^ found increased FC in schizophrenia patients between the areas of the right temporal pole and the left hippocampus.

One of the advantages of the present study is its naturalistic prospective design that renders a high ecological validity. Nevertheless, there are also some important limitations. The entire patient sample did not undergo an identical antipsychotic treatment, and even though this did not bias the rs-fMRI data (obtained before treatment onset), it may have had an effect on treatment response. Furthermore, the patient group classified as treatment responders had a significantly higher PANSS score and, hence, more severe psychotic symptoms at baseline, in comparison with the TRS patient group. Similar findings have been reported elsewhere^105^ and support the idea that current antipsychotic treatment options are more effective for more severe, pan-symptomatic patients. Nevertheless, this difference raises the possibility that the severity of the symptoms could be a confounding factor, explaining the differences in the neuroimaging results. Due to the mathematical specifics of CCM, it is not possible to add covariates to the analysis to eliminate the possibility of confounding effects. This is a limitation of our study. Future studies should investigate whether the observed neural differences can be explained by differences in schizophrenia symptom severity, as well as whether they are related to specific subtypes of FEP and whether they are related to specific types of symptoms. Moreover, it is worth noting that our results may not generalize to patients who develop TRS over time, who may harbour different biological pathways. Despite these limitations, our results reveal a network of key brain regions that can be further explored in FC studies of TRS research. Future research in the field should move towards precision medicine approaches to individualized treatment of schizophrenia.^106^ This endeavour would require the merging of neuroimaging with big data and machine learning techniques in order to transition from between-group comparisons to inferences on the individual level.^38,107^

## Conclusion

In summary, TRS represents the most chronic form of psychotic illness, a personal tragedy with a high caregiver and socioeconomic burden.^13^ Schizophrenia patients resistant to antipsychotic treatment comprise the lowest community functioning performance amongst psychiatric conditions^12^ and often experience worse clinical outcomes^19^ when compared with treatment-responsive patients. The present results contribute to a growing body of evidence that portrays TRS as a singular neurobiological sub-type of schizophrenia with a distinctive neural FC signature even at illness onset, before starting antipsychotic treatment. Hence, it is not surprising that TRS patients respond to fundamentally different treatment regimens than treatment-responsive patients.^34^ The current results contribute to disentangling the intricate neural patterns of treatment resistance in order to elucidate key predictors of TRS. Integrating functional magnetic resonance imaging in the assessment of psychotic patients at the time of their first episode, in order to decide on a certain line of treatment or another, opens up very promising future avenues. Having prior knowledge of the response to antipsychotic treatment of patients creates several possibilities to improve clinical decision-making at an early stage of the disease and, consequently, to improve patients’ quality of life.

## Supplementary Material

fcae094_Supplementary_Data

## Data Availability

Available data can be shared for non-commercial research purposes, upon reasonable request to the authors.
