# Supplementary material for: Aberrant connectivity in the hippocampus, bilateral insula and temporal poles precedes treatment resistance in first-episode psychosis: a prospective resting-state functional magnetic resonance imaging study with connectivity concordance mapping
Source: Brain Commun. 2024 May 4;6(3):fcae094. doi: 10.1093/braincomms/fcae094 (PMC11069118; doi:10.1093/braincomms/fcae094)
Supplement: fcae094_Supplementary_Data [file fcae094_supplementary_data.pdf]

Supplementary table 1. Measurements and questionnaires used at baseline and after 12 months.

	Baseline (study entry)	12 months
Basic demographics	x	
DUP, age of onset	x	
Premorbid functioning	x	
Life style (exercise, diet)	x	
Substance use	x	
CLINICAL		
Hospitalization	x	x
Diagnosis, comorbidity	x	x
PANSS	x	x
CDSS	x	x
Antipsychotic treatments	x	x
Treatment adherence	x	x
SUMD	x	x
SIDE EFFECTS		
SAS	x	x
AIMS	x	x
BARS	x	x
UKU	x	x
BMI	x	x
FUNCTIONING		
SOFAS	x	x
RFS	x	x
NEUROCOGNITION		
Information	x	x
Arithmetic	x	x
Digit span	x	x
Digit symbol	x	x
Letter number span	x	x
Logical memory test	x	x
Verbal fluency	x	x
MWCST	x	x
CNI	x	x
BRAIN IMAGING		
structural- and resting-statefMRI	x	

Abbreviations: DUP=Duration of untreated psychosis; PANSS=Positive & Negative Symptoms Scale; CDSS=Calgary Depression Scale for Schizophrenia; SUMD=Scale to assess unawareness of mental disorder; SAS=Simpson Angus Scale; AIMS=Abnormal Involuntary Movement Scale; BARS=Barnes Akathisia Rating Scale; UKU=Udvalg for Kliniske Undersøgelser; SOFAS=Social and Occupational Functioning Assessment Scale; RFS=Role Functioning Scale; MWCST=Modified Wisconsin Card Sorting Test; CNI=Cambridge Neurological Inventory.
